# Effect of core stabilization exercises in addition to conventional therapy in improving trunk mobility, function, ambulation and quality of life in stroke patients: a randomized controlled trial

**DOI:** 10.1186/s13102-022-00452-y

**Published:** 2022-04-08

**Authors:** Wajeeha Mahmood, Hafiz Syed Ijaz Ahmed Burq, Sarah Ehsan, Basita Sagheer, Tahir Mahmood

**Affiliations:** 1 Department of Physical Therapy, Superior University, Lahore, Pakistan; 2 Lahore General Hospital, Lahore, Pakistan; 3 Riphah International University, Islamabad, Pakistan; 4 University of Lahore, Lahore, Pakistan; 5 Imran Idrees Institute of Rehabilitation Sciences, Sialkot, Punjab Pakistan

**Keywords:** Core stabilization, Exercise, Mobility, Quality of life, Range of motion, Stroke

## Abstract

**Background:**

Stroke is a major cause of disability with mainly affecting trunk mobility and function. The purpose of this study is to determine the effectiveness of core stabilization exercises versus conventional therapy on trunk mobility, function, ambulation, and quality of life of stroke patients.

**Design:**

Assessor blinded randomized control trial.

**Setting:**

Ibrahim polyclinic—Shadman, Ch Muhammad Akram teaching hospital-Raiwind, Rasheed hospital-Defence.

**Subjects:**

Chronic ischemic stroke patients.

**Intervention:**

Control group (n = 21) underwent conventional treatment for stroke for 40 min/ day, 5 times/ week for 8 weeks. Experimental group (n = 20) received core stability training for additional 15 min along with conventional treatment.

**Main measures:**

Main outcome measures were Trunk impairment scale (TIS), functional ambulation category (FAC), stroke specific quality of life (SSQOL) and trunk range of motion (ROM).

**Results:**

The differences between the control group and experimental group post-treatment were statistically significant for trunk impairment, functional ambulation, quality of life, and frontal plane trunk motion (p-value < 0.05) with higher mean values for core stabilization training. The frontal plane trunk mobility and rotation showed non-significant differences post-treatment (p-value > 0.05).

**Conclusion:**

This study concluded that core stabilization training is better as compared to the conventional physical therapy treatment for improving trunk impairments, functional ambulation and quality of life among patients of stroke. The core stabilization training is also more effective in improving trunk mobility in sagittal plane. This study is registered in Iranian Registry of Clinical Trials IRCT20210614051578N1 and was approved by the local research ethics committee of Riphah International University.

## Background

Stroke with long-term disabilities is one of the major causes of decreased trunk mobility and its functions [1, 2] Stroke is a disabling disease, affecting 16 million people annually worldwide. In Pakistan, there is a lifetime stroke prevalence of 19%. [3] Common impairments due to stroke are decreased strength and delay in activity of trunk muscles. It leads to problems in positional sense, sitting balance and ambulation of patient. Balance is highly associated with trunk symmetry and function [1, 4]. In stroke patients prognosis is highly dependent upon trunk function. Gait is dependent upon trunk muscle activity. Static and dynamic balance is prognostic factor for recovery in stroke as head control and movements of limbs provide strong impact on activities of daily living. Core stability exercises play key role in functional outcomes in stroke patients [5].

Decreased trunk musculature strength causes poor posture which in turn leads to functional disorders and dependency. Thus for functional ambulation rehabilitation is required for trunk mobility [6]. For stroke patients, it is important to identify those factors that limit participation restriction in daily activities. So that one can work on modifiable factors and make their life more independent. This knowledge can help physiotherapist to plan effective treatment protocol for the patient [7]. As stated in International Classification of Functioning, Disability and Health, 70.5% the of primary outcomes classified were in domain of activity and participation [8]. Quality of life is highly affected and a decrease in core muscular strength leads to the risk of fall towards the involved side. Fall was observed in 26–35% of patients post-stroke because of the impaired balance. So the core trunk stability is essential for improving gait, flexibility and balance which is compromised inn such cases. This can be done using unstable supports like a swiss ball, physioball, balance pad, and balance cushion. They increase functional performance of trunk, accuracy in repositioning and also enhance the thickness of trunk musculature and balance in stroke patients [9]. Studies have shown that in stroke, affected leg spent thirty three percent of step cycle in swing phase as compared to a healthy individual gait cycle. While eighty percent of gait cycle is spent in stance phase as compared to normal swing to stance ratio [10].

Due to the speed reduction, more energy is consumed during the gait cycle in stroke. Core muscle stability can improve this reaction time. Thus gait and balance are directly affected by core stability exercises [11]. Core trunk stability is defined as conjugating work of the pelvis, lumbar and hip region to provide stability to the vertebral column, by keeping the erect posture of the body during sitting, standing, walk and preventing any buckling of the spine in case of any perturbation [12]. The core trunk musculature is comprised of abdominals, paraspinal, hip joint muscles and pelvic floor muscles [13]. Co-contraction of transverses abdominis and multifidus is essential for the stability of the spine in static and dynamic balance. Stroke patients lack the selective control of muscles, due to which muscle activation is compromised in stroke patients leading to falls [14]. Core muscles are strengthened in athletes and patients with low backache as they provide stability to the spine. During inspiration, the diaphragm creates intra-abdominal pressure which creates spinal stability. This intra-abdominal pressure activates pelvic floor muscles and eccentrically abdominals to provide spinal stabilization and posture is improved [15]. Trunk muscle mobility and their activation is highly dependent upon alignment of pelvis, any of the biomechanical abnormality of pelvis can decline their activation [16]. In patients having stroke, assessment related to disability, functional limitation and impairment alone does not provide the complete impact of the disease. The factor like psychological assessment and the quality of life must be considered for a complete assessment of patient [17]. The quality of life of the patient is dependent upon his physical health, emotional status, and feeling of being a socially active person. World Health Organization defines the quality of life related to health of a patient as the perception of their place in life according to needs, expectations of their culture. Disability due to stroke decrease daily functional activities [18]. There is a vital decrease in the quality of life of 25% of stroke patients in the first three months due to a general decline in the health of patients. Physical disability, a deficit of movement control, sensory issues, visual disturbances along psychological and cognitive issues further decline the health-related quality of life of stroke patients. The stress of disease and negative effects on family post-stroke further decline quality of life in patients. So patient finds it difficult to perform his social roles in society [19] Better trunk function can enhance quality of life of stroke patients [1]. Stroke patients have long-term disabilities with physical, cognitive, and psychological issues. So the quality of life is quite affected in these patients. Stroke is now treated as a chronic disease with multiple health-related and social issues other than major mortality [20]. The purpose of the study is to determine the effectiveness of core trunk stability exercises in stroke patients regarding trunk function and mobility which lead to enhanced quality of life of the patient.

## Materials and methods

It was an Assessor Blinded Randomized control trial following the CONSORT Statement Guidelines. This study is registered in Iranian Registry of Clinical Trials IRCT20210614051578N1 and was approved by the local research ethics committee. Individuals with chronic stroke were recruited from Ibrahim polyclinic – shadman, Ch Muhammad Akram teaching hospital Raiwind, Rasheed hospital-Defence, from July 2018. The trial was completed in 8 weeks and a follow-up was taken in September 2018.

Trial design was parallel (1:1). Participants fulfilling the inclusion criteria were selected consecutively and were then allocated randomly to the control group and Experiment group. The process of allocation was concealed from the participants and the researcher. It was performed by a research assistant who was not involved in any further step of research. To take readings at pre-treatment and post-treatment levels, outcome assessors were recruited, who were blinded to the treatment group.

Sample size was calculated using data from previous studies using trunk impairment scale a an outcome measurement tool with µ1−µ2 [1] the sample size was calculated using following formula.

$${\text{n}} = \frac{{\left[ {\left( {{\text{z}}_{{\alpha /{2}}} + {\text{ z}}_{\beta } } \right)^{{2}} {\text{x}}\{ {2 }\left( \sigma \right) \, \} } \right]}}{{\left( {\mu {1} - \mu {2}} \right)^{{2}} }}$$

where n is sample size required in each group. Z _α/2_ depends on level of significance, for 5% this is 1.96. Z_β_ depends on power, for 80% this 0.84 [21]. σ is standard deviation which is 3.33. µ1is mean change in trunk impairment scale values in experimental group which is 4.13. µ2 is mean change in trunk impairment scale values in conventional group that is 1.19 [1]. Based on the above formula, the sample size required per group is20. Hence total sample size required is 40. Considering a drop-out rate of 10% total sample size required is 44. (22 in each group).

The sample was recruited through consecutive sampling techniques and then randomly assigned to the control and experimental group through a random number table [22].

Both male and female participants with a history of first-time stroke were eligible for the study. Patients who have a chronic ischemic stroke (more than 6 months), not more than 1 year; with age group 45–65 years of age [23]; who have achieved sitting for at least 10 s and had a definite diagnosis of stroke confirmed through CT or MRI were included. They were excluded if they had severe cognitive and communication disorders that can affect interaction with a clinician, any neurological and sensory disorders other than stroke that can affect postural control, any visual, sensory, and hearing impairment that was not corrected through aids, any metabolic and malignant disorder which can lead to the emergency condition. Baseline readings were taken by a single investigator. Trunk impairment scale (TIS) was used to measure trunk performance in patients with neurological diseases. The reliability has been found to be 0.82 [24]. Trunk impairment scale evaluates static and dynamic trunk balance and trunk coordination. The scores for subscales of static sitting balance, dynamic sitting balance and trunk coordination are 7, 10 and 6 respectively. Total score ranges from 0 to 23, the higher the score the better the trunk performance [25]. A functional ambulation category scale was used to measure the mobility of the trunk. It consists of six categories and is scored from 1 to 6 where ‘1’ is for ‘non-functional’ and ‘6’ is for ‘independent’ [26].

Quality of life was measured using the Stroke-specific quality of life scale (SS-QOL). The scale consists of 12 domains in which the quality of life is determined. There are a total of 49 items in 12 domains. The domains are energy, family roles, language, mobility, mood, personality, self-care, social roles, thinking, upper extremity function, and vision and work productivity. The total score ranges from 49 to 245 with a higher score indicating a better quality of life and function. It is a valid and reliable tool to measure self-reported quality post-stroke (α ≥ 0.73) [27]. Trunk range of motion was measured using measuring tape and goniometer. Tape measurement is reliable in measuring the trunk range of motion (0.77–0.98) [28].

A sample size of 41 patients was taken with 21 patients in control and 20 patients in the experimental group. Patients were enrolled if they fulfilled inclusion criteria after proper and informed consent. They had the right to withdraw from the study whenever they want.

### Control group

Demographic data was noted. Patients were randomly divided through random number table. In control group patients were given conventional physical therapy 40 min/ day, 5 times/ week for 8 weeks including basic activity like range of motion exercises, mobility exercises like transfers and gait training [29], stretching, tone reduction [12], task oriented training and compensatory approach [1]. Task-oriented training emphasized the hemiplegic side. Goals were set to enhance mobility. For the upper limb, different objects were placed on the table, and the patient was asked to use them. These activities were performed for ADL training. For example, combing, brushing, eating activities were performed. Reaching and grasping activities were taught in a sitting position. Mat activities were performed with special emphasis on sitting positing.

## Experimental group

In the experimental group, core stability training was done along with conventional therapy for additional 15 min with progression in training by increasing the frequency of repetition from 10 to 20 repetitions according to patient condition. Core stability consisted of abdominal drawing-in maneuver (ADIM) for contraction of transverses abdominis [23].

After training of ADIM movements of the pelvis with and without ADIM were performed. Pelvic control exercises comprise anterior–posterior tilt, lateral shift, and transverse rotation. Exercises are performed in an upright posture [1]. Bed exercises were bridging exercises. Progression were made from bridging with both legs to one leg and then side bridging [30]. Curl up was done with progression from straight reaching, with diagonal reaching and with arms crossed [13]. Multifidus was also activated with the patient in a quadruped position and was asked to raise his arms alternatively. Then he extended his legs alternatively. Side bridging was performed to activate internal, external obliques and quadratus lumborum. Patients performed curl-ups for 15 degrees in crook lying position and maintained this position for 10 s [23]. The trunk range of motion was measured through measuring tape and goniometry. For thoracolumbar flexion 10 cm or four inches is considered an average measurement. It was measured by marking the spinous processes using a marking pencil of the C7 and S2 vertebrae as the subject was in a standing position. The posterior superior iliac spine and S2 spinous process are at the same level in the horizontal plane. The first measurement was taken when the patient was in a standing position. The second measurement was taken when the patient was in a flexed position. The difference between the two measurements was taken as thoracolumbar flexion range of motion.

For thoracolumbar extension C7 and S2 vertebrae, spinous processes were marked. The measuring tape was placed from the spinous processes of these two vertebrae in a standing position. The first reading was taken and then the patient was asked to extend his spine. Another reading was taken. The difference between the two readings was considered as a thoracolumbar extension. For lateral flexion to right and left side patient was asked to stand with feet shoulder-width apart with cervical, thoracic and lumbar spine in a neutral position. The spinous processess was marked between C7 and S2 vertebrae; the fulcrum of the goniometer was placed over the spinous process of S2 vertebrae. The proximal arm was placed perpendicular to the ground and the distal arm was aligned with the posterior surface of the spinous process of C7. Normally the value is 35 degrees to each side.

To check thoracolumbar rotation, the fulcrum of the goniometer was placed over the cranial aspect of the patient’s head. The patient is asked to rotate to one side. The proximal arm was placed parallel to the imaginary line between two prominent tubercles on iliac crests. The distal arm was placed parallel to an imaginary line between two acromial processes. The normal value is 45 degrees [31, 32]. Two readings were taken. One before treatment and the other after treatment of 8 weeks. Each patient received 5 treatment sessions per week for 8 weeks. The patient in the control group received 40 min treatment session while the experimental group received additional 15 min. Data collection performa included demographic data, outcome measurement tools Trunk Impairment Scale (TIS), Functional ambulation categories (FAC), Stroke Specific Quality of Life Scale (SS-QOL), and trunk range of motion through goniometry.

## Data analysis procedure

Data entry and analysis were done using SPSS 21. Quantitative variables were represented by Mean ± SD. Qualitative variables were represented by using a frequency table. P-value ≤ to 0.05 was taken as significant. An Independent t-test was used to determine the differences between the control and experimental group. Paired sample t-test was used to determine within-group differences. Shapiro-Wilks test of normality and uniformity was applied to check distribution. The data were normally distributed with p-value > 0.05.

**Fig. 1:**
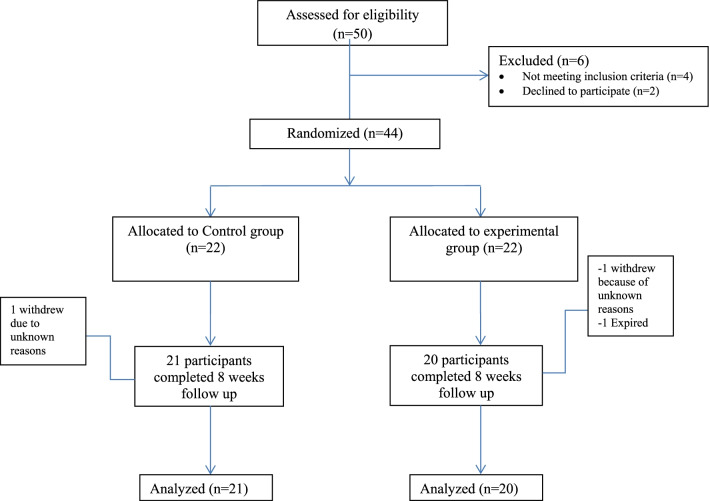
CONSORT Flow chart showing enrollment, intervention allocation and follow up of the patients

## Results

During the study period, 50 patients were assessed with chronic stroke, 4 were excluded due to ineligibility (Fig. 1) and two refused for participation. There were no crossovers between groups. Socio-demographic profile is described in Table 1. Males were more in control group than experimental group with right side more effected. Females were more in experimental group with left side was more affected. Both groups were comparable at baseline in terms of gender and side affected with stroke (p-value > 0.05). Both the groups had similarity in trunk impairment score, functional ambulation category, quality of life and trunk mobility at baseline (p-value > 0.05) in Table 2. There was statistically significant improvement between both the groups (P-value < 0.05) (Table 3). Quality of life was significantly improved with core stabilization training 161.90** ± **32.07 as compared to conventional therapy 124.95** ± **37.15. The improvement in trunk flexion and extension was statistically significant between both the groups (P-value < 0.05) with higher mean values post treatment in core stabilization group. However, trunk side flexion and rotation showed no significant differences between the two groups post-treatment (p-value > 0.05) (Table 3).

**Table 1:** Sociodemographic profile

	Control group (n = 21)	Experimental group (n = 20)	p-value
Age (years)	54.95 ± 6.35	57.10 ± 6.28	0.283
Gender			
Male	14	12	0.751
Female	7	8	
Effected side			
Right	12	9	0.538
Left	9	11

**Table 2:** Base Line measurement for trunk impairment scale, functional ambulation category, quality of life and trunk mobility

	Control group (n = 21)	Experimental group (n = 20)	p-value
	Mean ± SD	Mean ± SD	
Trunk impairment score	10.24 ± 4.6	10.5 ± 4.35	0.852
Functional ambulation category	3.43 ± 1.2	3.6 ± 0.94	0.598
SS-QOL	109.81 ± 40.0	111.80 ± 39.35	0.873
Trunk mobility			
Flexion	6.05 ± 2.29	6.00 ± 2.27	0.947
Extension	5.38 ± 1.80	5.30 ± 2.02	0.893
Side flexion right	11.04 ± 4.59	12.30 ± 4.92	0.405
Side flexion left	11.95 ± 4.08	11.45 ± 4.73	0.717
Rotation right	25.47 ± 5.2	25.65 ± 5.11	0.915
Rotation left	26.47 ± 5.45	25.25 ± 4.73	0.448

^*^SD = Standard deviation, **SS-QOL = Stroke specific quality of life

**Table 3:** Between group comparison of trunk impairment score, functional ambulation category, stroke specific quality of life, trunk mobility post-treatment

		Treatment group		p-value
		Conventional therapy (n = 21) (Mean ± SD)	Core stabilization training (n = 20) (Mean ± SD)	
Trunk impairment score	Pre-treatment	10.24 ± 4.6	10.5 ± 4.35	0.852
	Post-treatment	11.28 ± 4.50	15.01 ± 3.24	0.005
Functional ambulation category	Pre-treatment	3.43 ± 1.2	3.6 ± 0.94	0.598
	Post-treatment	3.91 ± 1.09	4.75 ± 0.85	.009
Quality of life	Pre-treatment	109.81 ± 40.0	124.95 ± 37.15	0.873
	Post-treatment	111.80 ± 39.35	161.90 ± 32.07	.002
Flexion	Pre-treatment	6.05 ± 2.29	6.00 ± 2.27	0.852
	Post-treatment	6.81 ± 2.18	8.40 ± 1.79	0.015
Extension	Pre-treatment	5.38 ± 1.80	5.30 ± 2.02	0.893
	Post-treatment	6.33 ± 1.79	7.85 ± 1.66	0.008
Right side flexion	Pre-treatment	11.04 ± 4.59	12.30 ± 4.92	0.405
	Post-treatment	12.09 ± 4.04	14.40 ± 3.84	0.069
Left side flexion	Pre-treatment	11.95 ± 4.08	11.45 ± 4.73	0.717
	Post-treatment	12.43 ± 3.82	14.45 ± 2.87	.064
Right side rotation	Pre-treatment	25.47 ± 5.2	25.65 ± 5.11	0.915
	Post-treatment	26.43 ± 4.71	29.05 ± 4.01	.063
Left side rotation	Pre-treatment	26.47 ± 5.45	25.25 ± 4.73	0.448
	Post-treatment	27.62 ± 5.17	29.45 ± 4.41	0.231

SD* = standard deviation

For within group comparison for Trunk impairment, Functional ambulation category, Stroke specific quality of life, Trunk Flexion Range, Trunk Extension Range, Trunk Right Side Flexion, Trunk Left Side Flexion, Trunk Right side rotation Range, Trunk left side rotation Range with mean differences were statistically significant for both the groups (p-value < 0.05) with greater difference seen in core stabilization training group (Table 4).

**Table 4:** Trunk impairment score, Functional ambulation category, Stroke specific quality of life, Trunk mobility across Control group and experimental group

Study group		Paired differences					T	df	p-value
		Mean	SD	SE Mean	95% CI of the difference				
					Lower	Upper			
Conventional therapy (n = 21)	TIS post treatment—TIS pre treatment	1.05	0.49	0.11	0.82	1.27	9.65	20	.000
Core stabilization training (n = 20)	TIS post treatment—TIS pre treatment	4.50	1.43	0.32	3.83	5.17	14.05	19	.000
Conventional therapy (n = 21)	FAC post treatment—FAC pre treatment	0.48	0.51	0.11	0.24	0.71	4.26	20	.000
Core stabilization training (n = 20)	FAC post treatment—FAC pre treatment	1.15	0.81	0.18	0.77	1.53	6.32	19	.000
Conventional therapy (n = 21)	SS-QOL post treatment—SS-QOL pre treatment	15.14	10.76	2.35	10.24	20.04	6.45	20	.000
Core stabilization training (n = 20)	SS-QOL post treatment—SS-QOL pre treatment	50.10	26.45	5.94	37.72	62.48	8.47	19	.000
Conventional therapy (n = 21)	Flexion post treatment—flexion pre treatment	0.76	0.43	.09	.56	.96	8.00	20	.000
Core stabilization training (n = 20)	Flexion post treatment—flexion pre treatment	2.4	1.18	.26	1.84	2.95	9.03	19	.000
Conventional therapy (n = 21)	Extension post treatment—extension pre treatment	.95	0.66	0.14	0.65	1.26	6.5	20	.000
Core stabilization training (n = 20)	Extension post treatment—extension pre treatment	2.55	0.68	0.15	2.22	2.87	16.16	19	.000
Conventional therapy (n = 21)	Right side flexion post treatment—right side flexion pre treatment	1.05	0.97	0.21	0.60	1.49	4.93	20	.000
Core stabilization training (n = 20)	Right side flexion post treatment—right side flexion pre treatment	2.10	1.51	0.33	1.38	2.81	6.18	19	.000
Conventional therapy (n = 21)	Left side flexion post treatment—left side flexion pre treatment	0.47	0.51	0.11	0.24	0.71	4.26	20	.000
Core stabilization training (n = 20)	Left side flexion post treatment—left side flexion pre treatment	3.00	2.17	0.48	1.98	4.01	6.16	19	.000
Conventional therapy (n = 21)	Right side rotation post treatment—right side rotation pre treatment	0.95	0.80	0.18	0.58	1.32	5.42	20	.000
Core stabilization training (n = 20)	Right side rotation post treatment—right side rotation pre treatment	3.4	1.73	0.39	2.59	4.21	8.79	19	.000
Conventional therapy (n = 21)	Left side rotation post treatment—left side rotation pre treatment	1.14	0.73	0.16	0.81	1.47	7.20	20	.000
Core stabilization training (n = 20)	Left side rotation post treatment—left side rotation pre treatment	4.20	1.15	0.26	3.66	4.73	16.31	19	.000

SD* = standard deviation, SE** = Standard Error, CI*** = confidence interval

## Discussion

Current study hypothesized that Core stabilization training is better in improving trunk impairment, functional ambulation, quality of life and trunk mobility as compared to conventional therapy in post stroke patients. Postural control is considered a vital component for maintaining an upright posture during stroke rehabilitation.The set of core stability with standard physical therapy exercises of 5 days a week for 4 weeks has beneficial effects compared to standard physical therapy alone among hemiplegic stroke patients, despite of small sample size was studied. But significant effects in experimental group were reported increased muscle activity lower trunk with a P value < 0.05 [14].

Current study stated that core stabilization training group had superior effects compared to conventional therapy alone with statistically significant differences (p-value < 0.05). These results are consistent with the results of a previous study conducted on determining the effect of core training on activity and stability of core muscles. It was reported that the experimental group i.e. the one receiving the core stabilization training showed statistically significant differences in the trunk impairment score (p-value < 0.05) [14].Core stabilization exercises have shown to improve the ambulation and gait parameters including step and stride length, cadence and velocity among s patients of stroke according to previous studies. Core stabilization exercises lead to increase in gait velocity of stroke patients [13].

Current study showed statistically significant improvement in functional ambulation in the core training group after treatment (p-value < 0.05). These results are consistent with the results of previous studies that showed the positive association of trunk muscle training and gait/ambulation. Stroke has a profound effect on the quality of life of the patients as it affects all the domains of life. It has been reported that post stroke depression has a significant association with the quality of life post stroke [33].

Literature has shown that various predictors of quality of life after stroke are age, sex, type of stroke, effected side, duration post stroke, severity of stroke, functional status, upper extremity and lower extremity motor function, balance, cognitive function, and depression [34]. Current study showed that there was significant improvement in quality of life of the patients post treatment with core stabilization training as compared to conventional therapy (p-value < 0.05). These results are supported by previous studies which irrespective of the type of exercise have reported that physical rehabilitation has a significant effect in improving quality of life post stroke [35]. It has been reported that home based exercises done immediately after stroke reduce the level of post stroke disability and also improve the quality of life [36].

In normal gait cycle, there is a coordination of movement between upper and lower trunk and they move in opposite directions about the vertical axis. For healthy individuals, the sagittal plane trunk flexion peaks close to every heel strike and reaches the maximal Range of motion in frontal plane trunk flexion at toe off. It has been reported that trunk plays a vital role in hemiplegic gait. Trunk movements are significantly altered post stroke because of muscle imbalance. The trunk impairments are bilateral contrary to the extremities [37].

Results of this study showed that the sagittal plane mobility of trunk had statistically significant differences between the two groups (p-value < 0.05). The core stabilization training group showed higher mean flexion and extension values post treatment as compared to conventional training group. But the results of a meta-analysis are inconsistent with our results. It reports that there is moderate evidence for effect of specific trunk exercise as compared to conventional therapy in early stroke rehabilitation, significantly in improving balance and trunk mobility post stroke. There was weak evidence for the effect of trunk exercises in improving trunk performance and function [38]. But Fujita et al. (2014) reported that even with mild stroke the weakness of abdominal muscles can occur, which has adverse effects on ADLs of the patients including their dress up, transfer and walk as their functional independence measure was lower in patients having weakness of abdominal muscles. Trunk specific training to overcome these abdominal muscles, was found effective in improving the activities of daily living [39]. Trunk exercises include specific upper and lower trunk movements in supine and sitting positions using stable/unstable support surfaces. Specific trunk exercises in addition to the standard physiotherapy treatment have been reported to be beneficial in improving trunk function in early stages of stroke [9].

Impaired trunk function is characterized by reduced sitting balance, decreased in trunk coordination, reduced trunk control and core muscle strength, and altered trunk position sense. Stroke Patients have shown increased frontal plane movements, but a reduction in sagittal plane movements compared to healthy controls. Core stability training along with sitting and reaching training have been found to improve static and dynamic sitting and standing balance post stroke [40]. In current analysis, core training groups had statistically significant difference in post treatment values of Trunk impairment, functional ambulation, quality of life and sagittal plane trunk mobility (p-value < 0.05). The reason behind can be the core stability exercises has induced positive impact that has improved their muscle activation, strength and functions. As reported that selective trunk exercises combined with functional exercises help in improving mobility, balance and trunk control in chronic stroke patients. Clinical observations have suggested that it is difficult to retrain the lower trunk side flexion and rotation movements in patients with chronic stroke [41]. Current study has shown that the side flexion and rotation did not have statistically significant difference between conventional training group and core stabilization group (p-value > 0.05). However, within group analysis showed that the differences in pre and post treatment mean ranges were statistically significant across the groups. But in contrast to Rai et al. (2014) reports that trunk rehabilitation and balance training were effective in improving trunk control, balance and gait. The improvement was better in the patients who received trunk specific training and balance training along with conventional physical therapy as compared to conventional treatment alone [42].

Novelty of this study is that previously no comparative study has been performed to study the effect of core stability exercises, trunk mobility and quality of life in stroke patients. This study will help to determine effect of trunk stabilization exercises on trunk function which is a strong predictor of ambulation. Hence results from this study will directly help to improve quality of life in patients. The main limitation of this study was that it could not investigate the long-term effects of core stabilization training and conventional therapy. It is recommended that further studies should be conducted with a larger sample size and follow-up assessments to ensure the generalizability of the study. The long-term effects of core stabilization training should also be studied with equal treatment intensity in both groups.

## Conclusion

This study concludes that core stabilization training is better as compared to the conventional physical therapy treatment in improving trunk impairments, functional ambulation and quality of life of stroke patients. The core stabilization training is also more effective in improving sagittal plane trunk mobility. Both conventional therapy and core stabilization training had similar effects in improving frontal plane trunk mobility and trunk rotation.

## Data Availability

The datasets used and/or analyzed during the current study are available from the corresponding author on reasonable request.
